# Cardiopulmonary Resuscitation Training by Avatars: A Qualitative Study of Medical Students’ Experiences Using a Multiplayer Virtual World

**DOI:** 10.2196/games.6448

**Published:** 2016-12-16

**Authors:** Johan Creutzfeldt, Leif Hedman, Li Felländer-Tsai

**Affiliations:** ^1^ Department of Clinical Science, Intervention and Technology (CLINTEC) Karolinska Institutet Stockholm Sweden; ^2^ Center for Advanced Medical Simulation and Training Karolinska University Hospital Stockholm Sweden; ^3^ Department of Psychology Umeå University Umeå Sweden

**Keywords:** avatars, cardiopulmonary resuscitation, educational technology, medical students, experiences, multiplayer virtual worlds, patient simulation, virtual learning environments

## Abstract

**Background:**

Emergency medical practices are often team efforts. Training for various tasks and collaborations may be carried out in virtual environments. Although promising results exist from studies of serious games, little is known about the subjective reactions of learners when using multiplayer virtual world (MVW) training in medicine.

**Objective:**

The objective of this study was to reach a better understanding of the learners’ reactions and experiences when using an MVW for team training of cardiopulmonary resuscitation (CPR).

**Methods:**

Twelve Swedish medical students participated in semistructured focus group discussions after CPR training in an MVW with partially preset options. The students’ perceptions and feelings related to use of this educational tool were investigated. Using qualitative methodology, discussions were analyzed by a phenomenological data-driven approach. Quality measures included negotiations, back-and-forth reading, triangulation, and validation with the informants.

**Results:**

Four categories characterizing the students’ experiences could be defined: (1) Focused Mental Training, (2) Interface Diverting Focus From Training, (3) Benefits of Practicing in a Group, and (4) Easy Loss of Focus When Passive. We interpreted the results, compared them to findings of others, and propose advantages and risks of using virtual worlds for learning.

**Conclusions:**

Beneficial aspects of learning CPR in a virtual world were confirmed. To achieve high participant engagement and create good conditions for training, well-established procedures should be practiced. Furthermore, students should be kept in an active mode and frequent feedback should be utilized. It cannot be completely ruled out that the use of virtual training may contribute to erroneous self-beliefs that can affect later clinical performance.

## Introduction

New generations of students, having grown up playing videogames and computer games, embrace these technologies for learning [1,2]. It has been shown that videogames used for entertainment may possess a transferable effect to medical skills [3]. Although several studies within diverse educational areas find serious games to be of benefit, more critical analyses of the effectiveness of computer-based serious games have questioned this by pointing out ambiguities and a need for further research [4,5]. In a review of serious games designed for health care professionals, Wang et al found positive training effects [6]. However, effects were hard to compare and heterogeneities and methodological difficulties were noted [6].

Several reports on benefits of this learning tool exist in the medical field [7-10]. Furthermore, skills and procedures taking place in group settings (as is often the case in the medical field) may potentially utilize training in multiplayer virtual worlds (MVWs) [11,12]. In contrast to so called *virtual patients*, MVWs enable training for teamwork skills, such as team coordination and team communication.

Traditionally, cardiopulmonary resuscitation (CPR) training is performed on mannequins under instructor supervision, focusing on single-person psychomotor skills, but in the last two decades alternative forms of basic CPR training have been introduced [13-15], including CPR team training using MVWs [16].

Studies of serious games would benefit from moving the focus away from the acquisition of CPR knowledge and skills, and instead focus on how students think and feel during MVW-CPR training. Furthermore, this approach may also indicate potential uses of MVWs and give rise to new questions about the use of MVWs for medical education. By using qualitative research methodology, new knowledge will be generated about this phenomenon, contributing to a novel and rapidly developing educational field. The aim of this study was to explore medical students’ experiences using MVW-CPR in groups via broad qualitative analyses.

## Methods

After regional research ethics committee approval, 12 first year medical students at Karolinska Institutet (Stockholm, Sweden) were recruited to the study. Inclusion criteria included elementary computer knowledge and previous CPR training in medical school. Exclusion criteria were restricted to any students who had previously used a virtual world-based serious game. To elucidate how virtual world training in groups was conceived, the students were invited to share their thoughts and experiences in focus group discussions.

The study was an after-training follow-up in which the trainees served as informants. Convenience sampling was used for this study. All subjects were enrolled by answering an invitation distributed by email to all first semester medical students at Karolinska Institutet. Upon enrollment, written consent was obtained and confidentiality was guaranteed by the authors.

**Figure 1:**
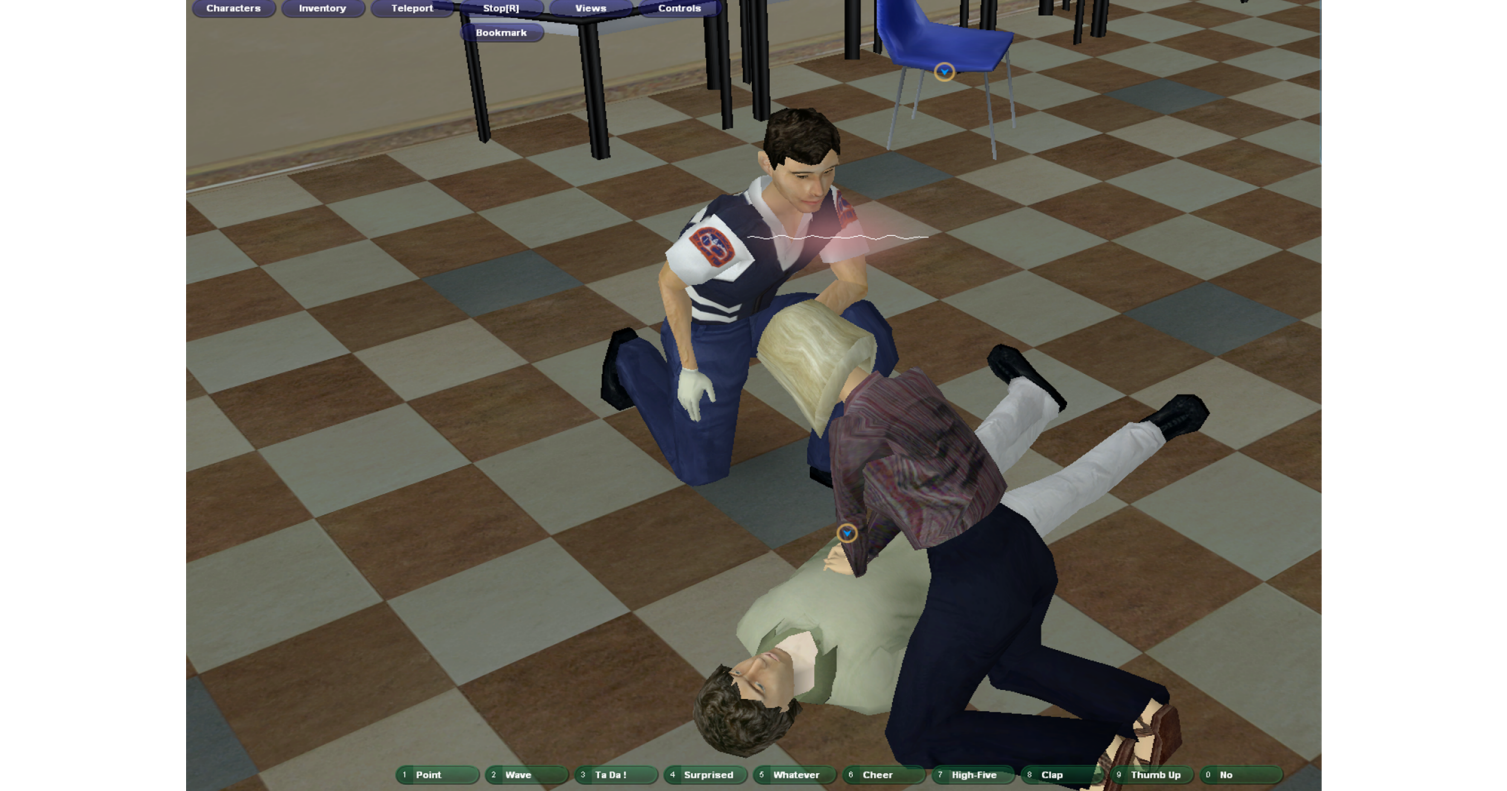
Interior of the multiplayer virtual world classroom, with a student (trainee) taking care of a victim as a paramedic arrives.

The virtual environment used during training (On-Line Interactive Virtual Environment) was developed in conjunction by the authors, coresearchers at Stanford University Medical Media and Information Technologies, and game developers at Forterra, Incorporated (San Mateo, CA). The MVW included a school building (interior and exterior) and a parking lot (Figure 1), and was accessed by standard personal computers connected to a server on the Internet. The subjects interacted by use of their avatars and a headset (voice over Internet protocol). The avatars’ movements and actions were controlled by a keyboard and computer mouse. Conventional gaming commands were used. Some essential commands for examination and treatment were accessible on *action tab-lists* defined by situation. During MVW training, the computers were isolated to prevent users from overhearing each other.

Before engaging in the training, participants were introduced to the virtual world by a virtual world instructor (avatar) that aided the trainees with navigational and procedural software commands, and taught the participants how to communicate with others. Altogether, familiarization lasted for approximately 15 minutes, and was followed by four scenarios in which a team of three trainees were instructed to act upon the need of the situation. In all scenarios, there was a victim-avatar (controlled by the instructor) that collapsed. The first two scenarios took place in a classroom, in which a teacher collapsed due to a cardiac arrest. The students (trainees) witnessed the event and had to take action: circulatory arrest was to be diagnosed and CPR started in accordance with existing bystander-CPR guidelines [17]. These guidelines require the rescuers to quickly start CPR when appropriate, call for help after circulatory arrest has been confirmed, and to relieve the rescuers in order to maintain the effectiveness of chest compressions. The third and fourth scenarios occurred outside the school building in a parking lot, where a person close to of a group of students (trainees) collapsed. The participants had to perform in the same manner as described above. In addition to taking care of the victim, the participants were expected to run to a phone to call 911, and to guide the paramedics to the victim and give a brief report. The scenarios lasted for 5-7 minutes each and ended when an avatar-paramedic entered the scene. After each scenario the participants were reassembled to receive real-world oral feedback from the instructor. The feedback focused on adherence to the CPR algorithm and how to coordinate activities in the resuscitation team.

As part of the MVW-CPR training program, a second identical training session was undertaken after 6 months. A semistructured group discussion approach was used, in which the aim of the discussion was to explore how the subjects had experienced the virtual world training. The discussants were the three participants who had just trained in the MVW, and the moderator was the instructor (JC). The discussion started 10-to-15 minutes after the end of the second training session. Data was collected by use of an audio recorder, and the discussions lasted for 55-to-65 minutes. Additional notes were collected by the moderator.

An interview guide was used (Multimedia Appendix 1), focusing on the central question of experiences, but the spontaneous emergence of topics was allowed. Participants were encouraged to speak freely and at length, and the moderator’s role was to pose open questions, highlight inconsistencies, and follow up on ambiguities. A verbatim transcription of the audio material was performed, and validation of the transcriptions was made by comparing parts of transcripts and recordings (JC, LH). By examining the degree of novelty of information in the following discussions, saturation was reached after 4 focus discussions with 12 participants.

A qualitative methodology described by Malterud was used [18]. This approach aims to describe a phenomenon by generating descriptions, categories, models, or theories. The aim of the analysis was to get a broad picture of the experience of using MVW for CPR training among medical students. A phenomenological data-driven approach was used, including analytical reduction, distillation, and aggregation [18]. Transcriptions were read back-and-forth independently by two researchers (JC, LH) who were blinded to the identity of the subjects. Themes characterizing the transcripts were negotiated, and a matrix was created in which the identified meaning bearing units from the informants were arranged according to theme. To enable further decontextualizing, the themes were replaced by codes that were refined, split, and combined as the process of connecting the analysis to the transcriptions and notes continued. Categories evolved as end products. To assert quality in the evolving process of analysis, grouping, and structuring of the decontextualized material, discussions and negotiations took place between two authors (JC, LH). All discussions were in Swedish and quotes were translated to English by the authors. Seven informants representing different gaming backgrounds, sexes, and experiences during the training were consulted at a late stage of analysis to verify and comment on the results.

## Results

The participants are characterized in Table 1.

### Quantitative Data on Self-Efficacy, Mental Strain, and Concentration

Following the same protocol as a previous study, before-training and after-training assessments of the subjects’ level of self-efficacy (using a 5-item validated instrument) was performed, and the levels of mental strain and concentration were assessed during the training using questions from validated instruments [16]. These assessments revealed that the level of mental strain in general was low and stable (mean 22/100, standard deviation [SD] 19 during the first scenario; mean 20/100, SD 14 during the last scenario) and concentration was moderate (mean 60/100, SD 13 during the first scenario; mean 65/100, SD 18 during the last scenario). Self-efficacy ratings were high before training (mean 5.8/7, SD 0.8) and increased further after training (mean 6.3/7, SD 0.6; *P*<.001, Wilcoxon signed rank test). A theoretical triangulation against these quantitative data was carried out after the qualitative analysis.

**Table 1:** Background demographics of the subjects.

Characteristic	Total (N=12)	Male (n=6)	Female (n=6)
Age, mean (standard deviation)	22.7 (2.6)	23.3 (3.6)	22.2 (1.2)
Number with access to computer at home	12	6	6
Level of computer experience^a^, median (range)	2 (1-3)	2 (2-3)	2 (1-2)
Videogame and computer game use^b^, median (range)	1 (0-3)	2 (1-3)	0.5 (0-1)

^a^Level of computer experience was graded on a 0-to-3 Likert-type scale (0=none, 1=beginner, 2=experienced, and 3=very high or expert).

^b^The use of computer and video games was graded on a 0-to-5 Likert-type scale (0=none, 1=less than once a month, 2=once every second week, 3=once a week, 4=several times every week, and 5=every day).

### Qualitative Analysis

The following four categories evolved as end products: (1) Focused Mental Training, (2) Interface Diverting Focus From Training, (3) Benefits of Practicing in a Group, and (4) Easy Loss of Focus When Passive.

#### Focused Mental Training

The participants generally enjoyed the training. In particular they mentioned that MVW was a reasonable and good way to go through the procedure of CPR in their minds. Their focus was on when and how to perform CPR. Their perceptions of it being a cognitive training are illustrated by the following representative quotes:

…it makes you improve each time you repeat… it feels like a very good complement - you really want to do more times.

Woman, 21, little previous gaming experience

Yes sometimes you think about that, you know, thinking ahead in the game and well to be a step ahead, kind of…

Man, 30, moderate previous gaming experience

However, frequent comments indicated that realism was lacking in the scenarios. There was lack of physical realism (virtual versus real-world) and participants were unable to perform all parts of the CPR procedure hands-on. The participants also indicated that the level of mental stress, although not completely absent, was much lower than would be expected in a real-world CPR event, exemplified by:

Alright, it feels like I remember what we have done, but it doesn’t feel like training because it is so unrealistic to me.

Woman, 22, little previous gaming experience

… it is not, no emotional tension at all, possibly there is some mental challenge, you have to think about, at least a bit about, what there is to do, but it´s no, it’s no stress in that sense.

Man, 19, moderate previous gaming experience

#### Interface Diverting Focus From Training

All participating medical students could relate to videogames and computer games, but the level of gaming experience varied. Despite this variability, the interface to the virtual world and the quality of the virtual world received a great deal of attention, substantiated in the following discussion. Students with less videogame and computer game experience commented on this issue, and indicated that they were unfamiliar with how they should interact in the virtual environment and control the avatar. This lack of familiarity had several consequences: it made it difficult to control and navigate the avatar, resulting in negative reactions; and trainees unaccustomed to this technology felt more distant to what was going on in the virtual setting.

Because, you know, if you haven’t played computer games it would take like a week before you felt comfortable in these movements and where to look and how it works…

Man, 21, moderate previous gaming experience

I think I was a bit afraid to press the wrong button so I stood somewhat passive, so to test - nope, it didn’t work.

Woman, 20, little previous gaming experience

Conversely, students with more videogame and computer game experience demonstrated a tendency to criticize and compare the interface and the environment with previous experiences:

…exactly, I think that it can help when you can choose yourself, you know, build [an avatar] yourself a bit, it is quite fun.

Man, 20, large previous gaming experience

[An improvement would be] maybe just monitoring [on the computer screen] on how, how well or bad this person [the victim] feels, kind of.

Man, 22, large previous gaming experience

To find further support, a post hoc quantitative analysis of meaning-bearing units was performed, focusing on technical difficulties versus sex. In this analysis, female participants addressed their own experienced difficulties five times, whereas males never did. Males mentioned the importance of gaming skills, in general terms, to act in the virtual world a total of seven times. This concept was never mentioned by females.

#### Benefits of Practicing in a Group

MVW technology enables trainees to learn in team constellations. In all virtual world scenarios, the participants trained together in groups of three. The strengths with this concept arose repeatedly during the discussions, and could be broken down into two subgroups: Practicing a Team-Based Activity in a Group, and Training in a Group is More Engaging.

##### Practicing a Team-Based Activity in a Group

CPR is often carried out in team settings. In the virtual world, the trainees could perform group activities that resembled what could be expected in real life; they were able to communicate to inform, seek support, and make decisions. Some team aspects of the CPR guidelines could also be simulated (eg, starting resuscitation while someone else was calling for help, relieving each other during CPR):

It was really good because otherwise you would have sat down yourself and done everything, but now I have to think about who does what; should I do that now or… and that was much better. You learn to cooperate in a completely different way than when you just do it yourself, and then when you actually join other persons too which do the same thing you get stunned, [what] should I do now? But you learn how to divide the tasks, I think, in quite short time.

Woman, 21, little previous gaming experience

Yes cooperate, and it’s actually where the biggest problems lie… Moderator: And here the training contributed? Yes, maybe it can’t be trained in any other way than just like this.

Man, 22, large previous gaming experience

##### Training in a Group is More Engaging

Some participants declared that performing the training together with others increased their engagement and made the training feel more fruitful (eg, making it easier to suspend feelings of unreality, and prosper from feedback and support from peers during training):

When, when I was coached kind of, like what they told me, I liked it, [it] was good…Then, besides, I guess it was much more fun [working together].

Man, 21, moderate previous gaming experience

Well, you get a little more focused if there are two others watching and three others looking who know it [what to do].

Woman, 22, little previous gaming experience

#### Easy Loss of Focus When Passive

A large category of conversation focused on feeling engaged in the task during the virtual training. Direct statements, as well as many ideas on how to increase the sense of directedness, arose during the discussions. In general, the students felt most challenged in the beginning of the training. With the addition of similar scenarios, a common experience indicated that the training became repetitive and less demanding. When there was a demand for action the participants were engaged, but when they were less active, they easily lost focus and got bored. It was pointed out that the trainee having the most mentally-demanding tasks was more focused, whereas others (eg, awaiting the arrival of paramedics) quite easily lost the sense of engagement in the endeavor, exemplified by the following quotes:

…and before, when it is tedious, you think about completely different things that, well, have to do with life outside.

Man, 30, moderate previous gaming experience

I think it is boring when you get the task to wait for the ambulance because then you really notice how long time this takes.

Woman, 23, very little previous gaming experience

## Discussion

### Principal Findings

Using serious games for CPR training was a novel experience for all participants. Several benefits of this training tool proposed in the literature (eg, the focus of the cognitive part of the training and the added value of group practice) have also reoccurred among our four categories. However, our results also draw attention to other important properties of scenario-based MVW training when used for medical education.

The most important finding was the close relationship between the level of activation and degree of difficulty, and the level of engagement reported by the subjects. One of the commonly assumed strengths using serious games is the capacity to engage the *player* [11,19]. Our data support this, as all participants were positive about the experience and gave examples of their engagement in the virtual world training. This engagement could be an effect of the training tool itself, but might also be related to the seriousness and importance of the subject. Conversely, the inherent characteristic of this technology to give rise to engagement cannot be taken for granted, as pointed out by Choi and Baek [20]. Conceptual similarities exist between the construct of *engagement* and that of *flow*, as described by Csikszentmihalyi [21]. In his model of optimal experience, Csikszentmihalyi predicts a flow experience when the level of challenge and the level of personal skills are matched [21]. Based on our results, this model seems appropriate. The CPR scenarios were quite repetitive and the level of difficulty did not increase, so the subjects (presumably getting more skilled) tended to experience less challenge, and hence moved from a sense of anxiety and arousal towards one of control, relaxation, and boredom. This finding underscores the concept of *leveling* in gaming practice (ie, a progressive increase in difficulty to maintain challenge and motivation) that should also be considered in serious games.

Focusing on our psychometric data, mean concentration (a conceptually important component of *flow*) displayed a tendency to increase after the first training scenario. Triangulating these data with the students’ experiences could imply that part of the increase is to be ascribed to the increased proficiency of maneuvering in the virtual environment and overcoming technical difficulties.

The seemingly close relationship between activation and challenge with engagement might be more pronounced than that experienced in real-life learning. According to constructivist learning theories, as the learner gets more accustomed and proficient, he or she moves into more complex reasoning and deeper understanding [22]. Accordingly, this development might not be as pronounced in a virtual world, and engagement in a virtual world might be more dependent on novelty and challenge. The assumption that a virtual world offers a less rich environment, in which the altered perception in the learning space affects our ways of learning, should be further elucidated. Furthermore, the use of serious games for tasks that may be monotonous can be questioned, since one of the key features of this educational tool (*engagement*) may be lost.

During training, feedback on performance was mainly given by peers in the virtual world, and by an instructor in the real-world immediately after each scenario. Overall, this feedback was appreciated, but the participants asked for more direct feedback within the virtual world. The virtual environment provides possibilities that tantalize the user to test and vary (eg, actions, appearances, and surroundings). In particular, with such characteristics incorporated into the virtual world, it is not surprising that the participants, whether previously accustomed to computer games or not, seek immediate feedback. Serious games lacking such features might render less active experimentation, and possibly less engagement [20].

The training was characterized as being mainly cognitive. Participants were accustomed to traditional CPR training, and several trainees noted the lack of psychomotor skills training as an important insufficiency. During training, the level of mental strain was low to moderate; this was confirmed in the discussions, and lack of stress was seen as another unrealistic feature of the virtual world training. The low level of stress has the potential to create a more structured and optimal learning situation during the initial phase of training. However, without training in a stressful and complex environment, transfer to real-world CPR situations may be hampered.

Discussions with the participants demonstrated an attitude of high capability in bystander-CPR. This theme was reflected by an attitude of mastery and a demand for more complex and difficult scenarios; there were many creative suggestions for variation and increased difficulty. In a previous study, we found that medical students reported that a weaknesses of MVW-CPR was in the area of, “tasks too easy, more options wanted” [16]. These finding agree with the increase in self-efficacy beliefs. It can be argued that shifting from a clinical high-stakes environment to a simulated (at least in this case) low stakes training environment might alter self-efficacy beliefs. Such an effect would be predicted by Bandura’s ideas about cost of failure, in which increased social cost tends to lower self-efficacy beliefs more if performance does not meet the needs of the situation [23]. If situations shift from partly decontextualized learning and training to high-stakes clinical reality, problems could arise in terms of misconceived self-beliefs and attitudes. Based on this issue, some positive aspects of using virtual worlds for learning and training (eg, engagement and experimentation) might have a downside of inadequate attitudes about situational demands and individual capabilities, as discussed by Wang et al [6]. Taking such risks into consideration, using virtual worlds for training in medicine (and probably other high risk professions) should be addressed with caution, not only by the trainees, but also by teachers, instructors, managers, and developers.

Serious games have been mentioned as an attractive alternative for digital natives [19], although this argument has also been disputed [24,25]. In this study, all participants were between 20 and 30 years of age, and the level of previous videogame and computer game experience varied. There was no clear correlation between age and experiences in the virtual training environment. The participants that were most clear about the lack of realism in the MVW were among the youngest. Therefore, the common belief that younger people would ask for more (and more easily accept) learning in virtual environments, could be questioned. These attitudes are likely attributable to more factors than simply age alone.

One category highlighted in this study was related to the influences of the interface. Most participants held strong opinions about its effects. Sweden is a highly computer technology-developed country, and students are accustomed to using computers; the Internet is regularly accessed for private and educational purposes [26]. Despite this general trend, students that were less experienced with computer games often mentioned difficulties with navigation in the MVW, along with other technical problems, whereas students with more experience tended to be forgiving towards software deficiencies. Representatives of the latter group tended to compare the MVW-CPR training with previous gaming experiences, and provided suggestions about how to make the interface and software more compelling, immersive, and enjoyable. To some extent, the opinions of the less-experienced and more-experienced participants are contradictory. Making use of high-end technological solutions that create more options for interactions, with the goal of making the experience more realistic, complex, and unpredictable, could render a virtual world that takes longer to get familiarized with. Interest in technology and computer games will vary among different users in medical fields, so a balance between technical solutions and usability must always be considered [27]. Furthermore, in the partly-experimental setting of this study, technical support was readily accessible, and interest from the instructors was high. Using this kind of training tool in an average educational setting also emphasizes the need to keep such technologies manageable for the users.

General discussions about videogames and computer games indicate that sex differences is a common issue; females and males seem to be attracted to different game genres [28]. Males and females also spend different amounts of time in different computer-related activities. It has also been reported that videogames and computer games have different meanings to men and women [29,30], and that men and women are attracted by different features in virtual worlds [31]. Kron et al discovered sex differences concerning attitudes towards video games among medical students [1], although a simplistic gender view is questioned by others [32]. Addressing this issue was not an aim of this study, making it hard to draw any further conclusions from present work on this issue. However, there seem to be some differences between males and females in our findings. Among the subjects, more men had a history of using videogames or computer games: this likely explains the tendency for female participants to focus more on technical difficulties in the MVW. Conversely, this trend does not explain why the importance of the training (versus importance of the interface itself) was raised more often by the females. Male participants appeared to display a more relaxed attitude towards the seriousness of the topic, and showed an interest in discussing how the tasks could be made more challenging, and the technical solutions more appealing. This difference in the importance of CPR competence among future physicians is mirrored by our previous results that demonstrated differences in self-efficacy beliefs before virtual world training among males and females [16].

In medicine, insufficient teamwork skills have been identified as a common cause for suboptimal performance and harm [33-35]. This problem also seems to be true during CPR [36,37]. Our findings indicate that the participants appreciated the team focus, and the creation of an atmosphere of shared tasks and responsibilities seemed to be of importance for engagement in the scenarios. Not only was there a common belief in the strength of practicing a team endeavor in a team setting, but the students also considered it to be more fun, inspiring, and rewarding to train together. Inferring theories of situated learning [38], it is likely that positive experiences might occur due to the students learning with their peers. This matter, investigated by deNoyelles and Seo, might be of great importance for virtual world learning [39].

The participants trained in four short virtual world scenarios on two occasions. Several participants were aware of the rapid deterioration of CPR skills, and suggested MVW training as a good way to repeat and retrain. Distributed training using self-directed methods, such as a serious game technology, seems like an attractive option for retraining CPR.

Our group of informants consisted of a very homogenous group of early Swedish medical students, making it difficult to generalize our results. However, we believe that our findings can be applicable to other virtual world scenario-based training programs in medicine, in which clinically inexperienced (but at least moderately computer-experienced) users can train while reflective feedback is given. The strengths of this study lie in its novelty and ability to highlight certain characteristics of medical team training in virtual worlds, and how these MVWs are experienced. Methodologically we have also triangulated our results with psychometric process variables and previous results to reach better credibility [16].

Further investigations should involve studies of more heterogeneous groups, and include outcome measures focusing on behaviors and performance after training (*transfer*). Studies focusing on when and where virtual world training is particularly effective would also be of interest. The perceptions and experiences of instructors, teachers, and other stakeholders involved in this educational technology are also warranted. In our data there were many suggestions about how to create better and more challenging scenarios, and make use of more exciting technical solutions. Adding stressful elements, such as a real-time timing and scoring that reflect chances for successful resuscitation, unforeseen interruptions, or multiple emergency medical conditions might increase the experienced levels of stress, and could be included when the learner has completed the basic procedural and team-oriented steps. Technology is advancing quickly in this area, and it would also be interesting to understand which features would facilitate learning. There is a definite risk of being captured by the possibilities of this learning tool, and how it is used and varied among game developers. Instead, it is important to utilize experience-based and scientifically grounded knowledge in the field of learning psychology.

There are several limitations of this study. Our participants were 12 first year Swedish medical students that actively answered a call for participants. Adding more informants could have added more experiences and unraveled additional categories. The scenarios were all CPR-related and the virtual training environment was partly developed for this study, and lacked many features of real-world scenarios (eg, noises, bystanders). Although our study was designed for a rich input of data, the aim of understanding how virtual world team CPR training is experienced cannot be fully reached. Experiences depend on individuals’ subjectivity, how the teams are arranged, and the learning situation in which the study is performed.

### Conclusions

Four categories—*Focused Mental Training, Interface Diverting Focus From Training, Benefits of Practicing in a Group, and Easy Loss of Focus When Passive*—illustrate the phenomenon of virtual world team CPR training among medical students. In order to be successful, we suggest that the use of scenario-based virtual world team training should address how to actively engage users in the training of shorter, well-established behaviors, and focus on procedures that contain a high degree of group member interaction and feedback. Learning by use of MVW training occurs on several levels, and pedagogic validity should be examined when changing from traditional real-world training to that of a virtual world.

## Appendix

Multimedia Appendix 1 Interview guide for focus group discussions.

## Abbreviations

CPR: cardiopulmonary resuscitation
MVW: multiplayer virtual worlds
SD: standard deviation

## References

[1] Kron FW, Gjerde CL, Sen A, Fetters MD (2010). Medical student attitudes toward video games and related new media technologies in medical education. BMC Med Educ.

[2] Schwarz D, Štourač P, Komenda M, Harazim H, Kosinová M, Gregor J, Hůlek R, Smékalová O, Křikava I, Štoudek R, Dušek L (2013). Interactive algorithms for teaching and learning acute medicine in the network of medical faculties MEFANET. J Med Internet Res.

[3] Kolga SM, Hedman L, Enochsson L, Kjellin A, Felländer-Tsai L (2008). Transfer of systematic computer game training in surgical novices on performance in virtual reality image guided surgical simulators. Stud Health Technol Inform.

[4] Akl EA, Pretorius RW, Sackett K, Erdley WS, Bhoopathi PS, Alfarah Z, Schünemann HJ (2010). The effect of educational games on medical students' learning outcomes: a systematic review: BEME Guide No 14. Med Teach.

[5] Pasquier P, Mérat S, Malgras B, Petit L, Queran X, Bay C, Boutonnet M, Jault P, Ausset S, Auroy Y, Perez JP, Tesnière A, Pons F, Mignon A (2016). A serious game for massive training and assessment of French soldiers involved in forward combat casualty care (3D-SC1): development and deployment. JMIR Serious Games.

[6] Wang TJ (2011). Educating avatars: on virtual worlds and pedagogical intent. Teaching in Higher Education.

[7] Andrade AD, Bagri A, Zaw K, Roos BA, Ruiz JG (2010). Avatar-mediated training in the delivery of bad news in a virtual world. J Palliat Med.

[8] LeFlore JL, Anderson M, Zielke MA, Nelson KA, Thomas PE, Hardee G, John LD (2012). Can a virtual patient trainer teach student nurses how to save lives--teaching nursing students about pediatric respiratory diseases. Simul Healthc.

[9] Patel V, Aggarwal R, Osinibi E, Taylor D, Arora S, Darzi A (2012). Operating room introduction for the novice. Am J Surg.

[10] Wiecha J, Heyden R, Sternthal E, Merialdi M (2010). Learning in a virtual world: experience with using second life for medical education. J Med Internet Res.

[11] Oblinger Dg (2004). The Next Generation of Educational Engagement. J Interactive Media Educ.

[12] Yee N (2006). The demographics, motivations, and derived experiences of users of massively multi-user online graphical environments. PRESENCE Teleoperators Virtual Environments.

[13] Braslow A, Brennan RT, Newman MM, Bircher NG, Batcheller AM, Kaye W (1997). CPR training without an instructor: development and evaluation of a video self-instructional system for effective performance of cardiopulmonary resuscitation. Resuscitation.

[14] Owen H, Mugford B, Follows V, Plummer JL (2006). Comparison of three simulation-based training methods for management of medical emergencies. Resuscitation.

[15] Wik L, Myklebust H, Auestad BH, Steen PA (2002). Retention of basic life support skills 6 months after training with an automated voice advisory manikin system without instructor involvement. Resuscitation.

[16] Creutzfeldt J, Hedman L, Medin C, Heinrichs WL, Felländer-Tsai L (2010). Exploring virtual worlds for scenario-based repeated team training of cardiopulmonary resuscitation in medical students. J Med Internet Res.

[17] International Liaison Committee on Resuscitation (2005). 2005 international consensus on cardiopulmonary resuscitation and emergency cardiovascular care science with treatment recommendations. Part 2: adult basic life support. Resuscitation.

[18] Malterud K (2009). Qualitative methods in medical research: an introduction.

[19] Prensky M (2001). Digital game-based learning.

[20] Choi B, Baek Y (2011). Exploring factors of media characteristic influencing flow in learning through virtual worlds. Comput Educ.

[21] Csikszentihalyi M (2008). Flow: The Psychology of Optimal Experience (P.S.).

[22] Vygotsky Ls, Cole M (1978). Mind in society: the development of higher psychological processes, 14 edition.

[23] Bandura A (1997). Self-efficacy: the exercise of control.

[24] Margaryan A, Littlejohn A, Vojt G (2011). Are digital natives a myth or reality? University students' use of digital technologies. Comput Educ.

[25] Sanchez J, Salinas A, Contreras D, Meyer E (2011). Does the new digital generation of learners exist?. Brit J Educ Technol.

[26] http://www.internetlivestats.com/internet-users-by-country/.

[27] Dodds TJ, Mohler BJ, Bülthoff HH (2011). Talk to the virtual hands: self-animated avatars improve communication in head-mounted display virtual environments. PLoS One.

[28] Carr D (2005). Contexts, gaming pleasures, and gendered preferences. Simul Games.

[29] Broos A (2005). Gender and information and communication technologies (ICT) anxiety: male self-assurance and female hesitation. Cyberpsychol Behav.

[30] Hoeft F, Watson CL, Kesler SR, Bettinger KE, Reiss AL (2008). Gender differences in the mesocorticolimbic system during computer game-play. J Psychiatr Res.

[31] Choi G, Chung H, Kim Y (2012). Are stereotypes relative to gender usage applicable to virtual worlds?. Int J Hum Comput Interact.

[32] Jenson J, de Castell S (2010). Gender, simulation, and gaming: research review and redirections. Simul Games.

[33] Mills P, Neily J, Dunn E (2008). Teamwork and communication in surgical teams: implications for patient safety. J Am Coll Surg.

[34] Mazzocco K, Petitti DB, Fong KT, Bonacum D, Brookey J, Graham S, Lasky RE, Sexton JB, Thomas EJ (2009). Surgical team behaviors and patient outcomes. Am J Surg.

[35] Neily J, Mills PD, Young-Xu Y, Carney BT, West P, Berger DH, Mazzia LM, Paull DE, Bagian JP (2010). Association between implementation of a medical team training program and surgical mortality. JAMA.

[36] Hunziker S, Johansson AC, Tschan F, Semmer NK, Rock L, Howell MD, Marsch S (2011). Teamwork and leadership in cardiopulmonary resuscitation. J Am Coll Cardiol.

[37] Mäkinen M, Aune S, Niemi-Murola L, Herlitz J, Varpula T, Nurmi J, Axelsson AB, Thorén A, Castrén M (2007). Assessment of CPR-D skills of nurses in Göteborg, Sweden and Espoo, Finland: teaching leadership makes a difference. Resuscitation.

[38] Lave J, Wenger E, Harrison R, Reeve F, Hanson A, Clarke J (2002). Legitimate peripheral participation in communities of practice. Supporting lifelong learning.

[39] deNoyelles A, Seo KJ (2012). Inspiring equal contribution and opportunity in a 3d multi-user virtual environment: bringing together men gamers and women non-gamers in Second Life. Comput Educ.

