# Supplementary material for: Cardiopulmonary Resuscitation Training by Avatars: A Qualitative Study of Medical Students’ Experiences Using a Multiplayer Virtual World
Source: JMIR Serious Games. 2016 Dec 16;4(2):e22. doi: 10.2196/games.6448 (PMC5203677; doi:10.2196/games.6448)
Supplement: Multimedia Appendix 1 [file games_v4i2e22_app1.pdf]

Multimedia Appendix 1. Interview guide for focus group discussions.

Key question: How is the virtual world scenario based CPR team training perceived (concerning flow/concentration, engagement, technique and interaction with others)

	Theme	Question
1	Affective engagement	1a. What did you feel during the training?<br>1b. How engaged were you?<br>1c. Why were you (not) engaged?<br>1d. What would be needed to increase your level of engagement
2	Focus during training (concentration)	2a. What did you think about during the training?<br>2b. How easy/difficult was it to perform good CPR?<br>2c. Did you experience “flow”?<br>If yes: How often, how much?
3	Teamwork	3a. How did you experience working together in a team?<br>3b. Which importance did your peers in the group have when dealing with the task?
4	Interface	4a. How did you experience being represented and acting in a virtual world?<br>4b. Did it make any difference if you acted in a virtual world before? How?<br>4c. How was it for you to use this training tool? Name the most important pros and cons.
5	Effects on knowledge	5a. Do you think that the CPR that you practiced in the virtual environment can affect how you act in a real emergency medical situation?<br>5b. Did you learn anything from the training? What?
